# The Prevalence of Metabolic Syndrome in Iran: A Systematic Review and Meta-analysis

**Published:** 2018-04

**Authors:** Saeideh MAZLOOMZADEH, Zahra RASHIDI KHAZAGHI, Nouraddin MOUSAVINASAB

**Affiliations:** Zanjan Metabolic Diseases Research Center, Zanjan University of Medical Sciences, Zanjan, Iran

**Keywords:** Metabolic syndrome, Prevalence, Systematic review, Meta-analysis

## Abstract

**Background:**

Metabolic syndrome (MS) is a collection of metabolic disorders which leads to early cardiovascular disease and diabetes type II. Regarding the wide range of its prevalence in Iran, this systematic review and meta-analysis determined the overall prevalence of the metabolic syndrome in Iran.

**Methods:**

In this systematic review and meta-analysis, the Medline, ISI, IranMedex, and SID were searched using “metabolic syndrome”, “syndrome X”, “prevalence”, and “Iran” keywords from 2002 to 2012. A total of 223 articles were found in which 14 studies were considered for meta-analysis. Data were analyzed using fixed and random model and meta-regression in STATA.

**Results:**

The prevalence of MS for those who were 20 yr and older was 23.8% (95%CI: 18.99–28.67) and in under 20 was 10.98% (95%CI: 7.75–14.2). Metabolic syndrome was more frequent in women (25.5%) than in men (17.16%) and was increased with increasing age. The most frequent component of metabolic syndrome was low HDL cholesterol (59.7%) followed by hypertriglyceridemia (39.5%).

**Conclusion:**

Regarding a high prevalence of metabolic syndrome and some of its components such as low HDL and high triglyceride in our country, implementation of programs for metabolic syndrome prevention is necessary.

## Introduction

The metabolic syndrome (MS), also known as X syndrome, is a collection of metabolic disorders which leads to early cardiovascular disease and diabetes type II. Since 1988, the definition of MS has been modified (1–4). The definition provided by the Adult Treatment Panel III (ATP III) seems to be the most internationally used definitions (5), although, in 2009, the International Diabetes Federation (IDF) and the American Heart Association/National Heart, Lung, and Blood Institute standardized their criteria for defining MS (6).

The prevalence of MS is high in the USA and Europe. In the USA, people aged >=20 yr had an MS prevalence of 34% (7). A review of the results of 11 cohort studies conducted on non-diabetic people from a number of European countries indicated that the prevalence of MS was 15.7% in males and 14.2% in females (8). In Asian countries, the prevalence of MS was ranged from 10% to 20% (9).

There are a number of studies reported the prevalence of MS in Iran between 8% and 35% (10–13), however, the majority of these studies do not reflect the real prevalence of this syndrome among the general population. This may due to use of various methods including design, population and definition of MS in the studies.

This systematic review and meta-analysis was conducted to determine the overall prevalence of the metabolic syndrome in Iran.

## Methods

### Search strategy

We searched two English-language electronic databases, PubMed and Web of Science. The terms used to describe metabolic syndrome were taken from the PubMed (MeSH) dictionary. The keywords used were “metabolic syndrome”, “syndrome X”, “prevalence” and “Iran”. In addition, other databases in Persian language such as IranMedex and Scientific Information Database (SID) and Scientific Journals of Iran were searched using Persian keywords that were comparable to our English keywords. We hand-searched conference proceedings and reviewed bibliographies of retrieved publications with the same strategy. Searches were limited to articles published between Jan 2002 and Jun 2012.

### Study selection

Eligible studies were those that reported the prevalence of metabolic syndrome in a random sample of Iranian population with a sample size of equal or greater than 300. We included studies that defined metabolic syndrome based on NCEP/ATP III and revised ATP III criteria. Two investigators independently assessed the eligibility of articles.

First, we reviewed titles and abstracts of articles and if there were no sufficient information in a particular title or abstract, we proceeded to check the full text. Afterwards, the quality of studies was assessed according to STROBE checklist on title, abstract, introduction, methods, results, and conclusion (14).

### Data extraction

Two investigators independently extracted data from included studies. We recorded author and year of publication, number, and gender of participants, age range of the population, sampling method, date of publication, study location, the prevalence of metabolic syndrome and its components, as well as the definitions and criteria for metabolic syndrome and its components.

### Statistical analysis

Statistical heterogeneity was assessed using Cochran’s Q-test and I^2^ statistic. According to the heterogeneity test, significant variations were found between study findings. Therefore, random effect model was used to estimate the overall prevalence of metabolic syndrome. The point estimations and their 95% confidence intervals (CIs) were computed and presented in forest plots.

To assess the effect of age, sex and publication date as possible sources of the heterogeneity for study findings, meta-regression model was used. Using restricted likelihood method, tau-square (τ^2^) was estimated as the indicator of heterogeneity. All analyses were performed in STATA software version 9.

## Results

**Fig. 1:**
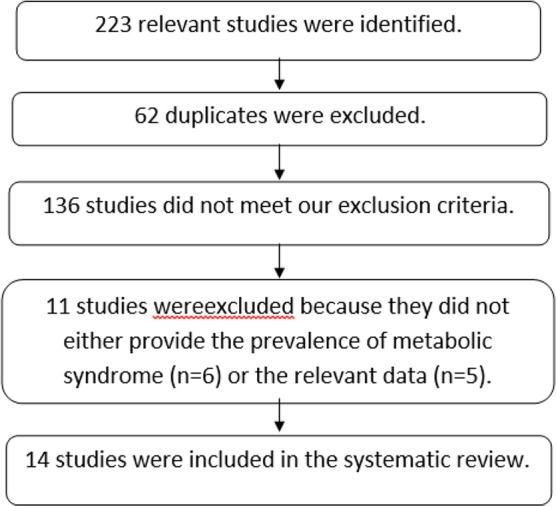
Flow diagram of Studies included in the systematic review

Overall, 223 studies were identified in our initial literature search. After screening the titles and abstracts of these publications, 62 duplicates were excluded and 161 studies were retrieved and assessed for eligibility. Finally, 14 were included in our systematic review (Fig. 1).

The remaining 147 studies were excluded either because they did not meet our inclusion criteria (n=136), they did not report the prevalence of metabolic syndrome (n=6) or their full texts were not available and they did not provide necessary data in the abstracts (n=5).

Of the 14 studies (n= 46464) included in our systematic review (10–13, 15–24), 11 were published in English and 3 in Persian. They had sample sizes ranging from 380 (in Shiraz) to 12514 (in Esfahan) patients (Table 1). The prevalence of the metabolic syndrome in these studies varied from 8.1% to 34.7%.

**Table 1:** Prevalence studies included in the meta-analysis

	***Reference***	***Place***	***Age (yr)***	***Sex***	***Sample size***	***Sampling method***	***Prevalence of MS***	***Definition***	***Year***
1	Sharifi et-al (15)	Zanjan	>20	Both	2941	Random	23.7	ATP III	2009
2	Delavar et-al (16)	Babol	30–50	Female	984	Random	31	ATP III	2009
3	Delavari et-al (13)	Tehran	25–64	Both	3024	Random	34.7	ATP III	2009
4	Kalishadi et-al (17)	Esfahan	6–18	Both	4811	Random	14	ATP III	2008
5	Esmaeilzadeh et-al (18)	Tehran	10–19	Both	3036	Random	10.1	ATP III	2006
6	Azizi et-al (12)	Tehran	>=20	Both	10368	Random	33.7	ATP III	2003
7	Jalali et-al (19)	Fars	19–90	Both	1402	Random	25.6	ATP III	2008
8	Kazemi et-al (20)	Zanjan	17–21	Both	507	Random	8.5	ATP III	2008
9	Ebrahimi et-al (21)	Shahreza	15–49	Female	1501	Random	9.7	ATP III	2007
10	Sarrafzadeghan et-al (11)	Esfahan	>=19	Both	12514	Random	23.3	ATP III	2008
11	K khayee et-al (22)	Zahedan	>=19	Both	1802	Random	21	ATP III	2011
12	Irvani et-al (10)	Shiraz	30–39	Male	380	Random	8.1	ATP III	2010
13	Rezaeeyanzadeh et-al (23)	Yazd	20–74	Both	2000	Random	21.3	ATP III	2005
14	Esmaeilnasab et-al (24)	Kurdestan	25–64	Both	1194	Random	29.1	ATP III	2005

**Fig. 2:**
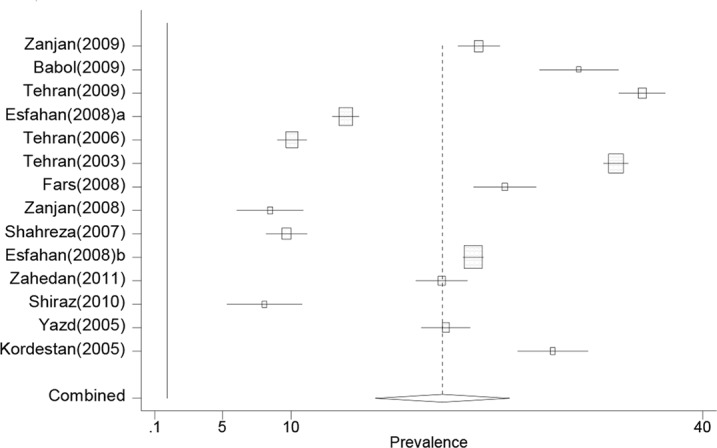
The prevalence of the metabolic syndrome

The overall prevalence of the metabolic syndrome based on these studies was 21.1% (95%CI: 16.2 – 25.9) (Fig. 2).

**Fig. 3:**
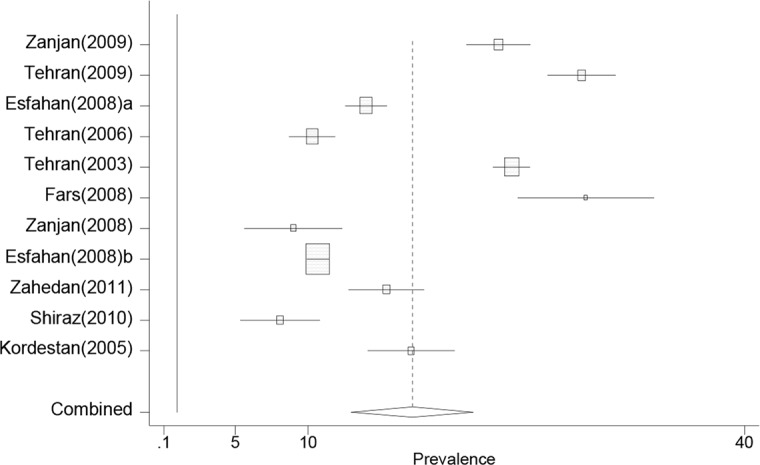
The prevalence of the metabolic syndrome in males

**Fig. 4:**
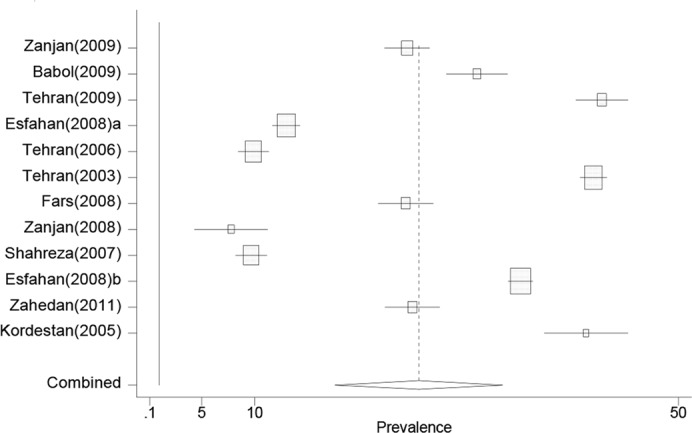
The prevalence of the metabolic syndrome in females

The prevalence of the metabolic syndrome was 17.2% (95% CI:13.0–21.3) in males and 25.5% (95% CI:17.6–33.4) in females, respectively (Fig. 3, 4). The prevalence of each of the 5 components of the metabolic syndrome was as follows: low HDL cholesterol, 59.7% (95% CI: 51.9–67.4); hypertriglyceridemia, 39.5% (95% CI: 31.9–47.1); central obesity, 38.9% (95% CI: 23.1–54.8); hypertension, 25.9% (95% CI: 17.1–34.8); and elevated fasting glucose, 16.5% (95% CI: 12.1–21.0).

Significant heterogeneity existed between studies (I^2^ = 99%, *P*<0.0001). The results of meta-regression showed that age was the main source of heterogeneity as with increasing one year in the mean age of subjects, the prevalence of metabolic syndrome increases 0.6% (*P*=0.047). However, sex and publication year had no effect on the estimated prevalence (*P*>0.05).

The age-stratified prevalence of the metabolic syndrome in subjects >20 yr was 23.8% (95% CI: 18.9 – 28.7). The prevalence of the metabolic syndrome in male and female subjects >20 yr were 19.5% (95% CI: 13.7–25.3) and 30.6% (95% CI: 22.4–38.8), respectively. The prevalence of low HDL cholesterol, hypertriglyceridemia, central obesity, hypertension and hyperglycemia in subjects over 20 were 58.9% (95% CI: 49.9–67.9), 41.8% (95% CI: 32.5–50.9), 46.1% (95% CI: 28.5–63.6), 29.9% (95% CI: 19.3–40.4), 19.4% (95% CI: 13.1–25.6), respectively.

The overall prevalence of the metabolic syndrome for those who were <20 yr old was 10.9% (95% CI: 7.8 – 14.2) and for male and female subjects <20 were 11.2% (95% CI: 8.2–14.2) and 10.5% (95% CI: 7.8–13.3), respectively. The prevalence of low HDL cholesterol, hypertriglyceridemia, central obesity, hypertension and hyperglycemia in subjects less than 20 were 62.3% (95% CI: 40.8–83.8), 31.4% (95% CI: 22.8–40.0), 12.7% (95% CI: 2.4–22.9), 11.6% (95% CI: 0.7–22.4), 5.7% (95% CI: 2.2–9.2), respectively.

## Discussion

This study revealed that at least one-fifth of population had MS in Iran. The overall prevalence of MS in Iran (21.1%) was lower than that reported in the USA (34%) (7), close to the prevalence of MS in some European countries (23%) (25), and was higher than that in studies from East of Asia (9, 26, 27). In a systematic review, in Latin America, the weighted mean prevalence of MS was 24.9% that was close to the prevalence of MS in our study (28). However, comparison of the results of these studies may not be straightforward because of the methodological differences among them. Even in our review, a wide range of MS prevalence was found among studies. Therefore, it would be a point of concern if we simply used meta-analysis methods to combine the findings of studies, even using random effect models. Since age was the main source of heterogeneity, we estimated the prevalence of MS for two age groups; less than 20 and more than 20 yr old separately to minimize heterogeneity. In addition, publication bias is one of the other issues considered in this study although we searched all available data sources to cover grey literature as much as we could.

The prevalence of MS was higher in women (25.5%) than in men (17.2%) in our study. No significant difference in the prevalence of MS between men and women was observed (28). However, in a systematic review, in Gulf Cooperation Council Countries, the prevalence of MS was higher in women (32.1%–42.7%) than in men (20.7%–37.2%) (29). In Australia, the prevalence of MS was higher in men than in women (30). The higher prevalence of MS in women in our study may be due to higher frequency of obesity and physical inactivity in Iranian women. Furthermore, the greater proportion of Iranian women are non-employed rather than employed, therefore they spend their days at home and thus might consume more foods.

In this study, the most frequent component of MS was low HDL cholesterol (59.7%) that was in accordance with the finding in Latin America (62.9%) (28). Although low HDL in Iran may be related to lifestyle changes such as unhealthy diet and physical inactivity, it may be due to genetic factors like mutation in the CETP locus and increased production of the hepatic lipase gene (19).

The second most frequent component of MS in our study was hypertriglyceridemia (39.5%) as it was in the Latin America study but with a lower proportion (46.7%) (28). The prevalence of central obesity and hypertension in our study were 38.9% and 25.9%, respectively, which was lower than that in other studies (29, 7, 31). The prevalence of elevated fasting glucose was lowest among components of MS in this study (16.5%). This finding was also reported by other studies conducted in China, Latin America, Spain, and Russia (26, 28, 31, 32). The differences in the prevalence of the components of MS among studies might be explained by genetic, environmental and sociodemographic factors in these countries or their populations.

In our study, the prevalence of MS increased with increasing age. This was also reported by other studies (26, 28, 31, 33, 34). The age-stratified prevalence of MS in subjects who were more than 20 yr was 23.8% and for those who were less than 20 yr old was 10.9%. In NHANES study in the USA, conducted on 2456 adolescents aged 12 to 19 years, the prevalence of MS was 8.6% (95% confidence interval, 6.5%–10.6%) and about half of the participants had at least one disordered measurement (35). In a study on 2761 adolescents aged 15 to 19 years, the prevalence of MS was 8.6%. The high prevalence of MS in our study might due to a high prevalence of obesity in our children (36). The most frequent component of MS was low HDL cholesterol (62.3%) in those who were less than 20 yr old as observed in other studies in Iran and Turkey that might reflect an ethnic predisposition toward this type of dyslipidemia in the region (37, 38).

## Conclusion

This study indicates a high prevalence of metabolic syndrome in Iran and its association with age. Some components of MS such as low HDL and high triglyceride were more common in our population compared to others. Therefore, this finding could contribute to the planning of prevention strategies to combat metabolic syndrome, as it is associated with a high risk of mortality and increased health-care costs for the Iranian government and population.

## Ethical considerations

Ethical issues (Including plagiarism, informed consent, misconduct, data fabrication and/or falsification, double publication and/or submission, redundancy, etc.) have been completely observed by the authors.
